# The Optimal Intervention Time of Bone Marrow Mesenchymal Stem Cells in Ameliorating Cardiac Fibrosis Induced by Viral Myocarditis: A Randomized Controlled Trial in Mice

**DOI:** 10.1155/2017/3258035

**Published:** 2017-12-05

**Authors:** Tingting Wu, Yuan Xie, Jing Huang, Ping Li, Xuliang Wang, Yaoyao Yan, Tianhe Xia, Lei Li, Feng Zhu, Hao Li, Rongzhou Wu

**Affiliations:** Children's Heart Center, The Second Affiliated Hospital and Yuying Children's Hospital, Institute of Cardiovascular Development and Translational Medicine, Wenzhou Medical University, Wenzhou, Zhejiang 325027, China

## Abstract

Bone marrow-derived mesenchymal stem cells (BMSCs) have recently been introduced to treat cardiovascular diseases, such as myocardial infarction and dilated cardiomyopathy. Nevertheless, there are few researches focused on the application of BMSCs in treating viral myocarditis, not to mention its optimal intervention timer potential mechanisms. In our study, we concentrated on finding an optimal time window to perform BMSCs treatment in a murine model of myocarditis induced by coxsackievirus B3 (CVB3). On the 1st day, 3rd day, 7th day, and 14th day after BALB/c mice were infected by CVB3, we intravenously injected equivalent BMSCs into the treatment groups. With a 28-day follow-up after inoculation, we found that the ventricular function was significantly improved in the BMSCs treatment group and cardiac fibrosis markedly ameliorated, especially when BMSCs were injected between 1 and 2 weeks after CVB3 inoculation. Furthermore, we demonstrated that after BMSCs treatment, the expressions of TGF-*β*, col1*α*1, and col3*α*1 were significantly decreased. Therefore, we conclude that BMSCs may have a potential to improve CVB3-induced myocarditis by ameliorating cardiac fibrosis through the inhibition of TGF-*β* expression.

## 1. Introduction

Viral myocarditis (VMC) is the inflammation of cardiac muscle which is mostly due to viral infection such as coxsackievirus B3 [1]. The disease is characterized by persistent inflammation and loss of cardiomyocytes which are later gradually replaced by fibrosis [2]. Although it is a self-limited infection in most patients [3], there are severe conditions, like sudden unexpected death and dilated cardiomyopathy (DCM) [4, 5]. Symptomatic treatments, for example, cardiac nutritional support, immunosuppressive drugs, and gamma globulin treatment, are still the main treatments, and there is hardly any therapy to prevent DCM [6]. Huang et al. [7] have detected a higher rate of apoptosis and necrosis in CVB3-induced myocarditis. And signals like TGF-*β*1 and other cytokines were sent to stimulate the growth of fibroblasts and the secretion of collagen fiber, which might cause dilated cardiomyopathy or even heart failure. Therefore, in the process of dilated cardiomyopathy for VMC patients, it is essential to ameliorate cardiac fibrosis.

Mesenchymal stem cells are pluripotent stem cells derived from mesodermal organs. They are widely found in the connective tissue and interstitial tissue of the whole body and especially rich in bone marrow [8]. Just like embryonic stem cells, BMSCs can self-renew and differentiate into osteocytes, chondrocytes, adipocytes, cardiomyocytes, and so forth [9]. Besides, BMSCs play an effective role in antiapoptosis, antifibrosis, angiogenesis, the renovation of damaged tissue, and immunoregulation [10]. With good safety and low immunogenicity, BMSCs are extensively studied in various diseases [11]. Moreover, recent studies discovered that MSCs enhanced the protective effects in myocardial infarction, DCM, VMC, and other cardiovascular diseases [12]. The mechanisms involved are not yet clear. Zhang et al. uncovered that BMSCs could secrete exosomes to promote proliferation, migration, and tube formation of cardiac stem cells [13]. It suggested that BMSCs might create a suitable microenvironment for cardiac stem cells to differentiate into cardiomyocytes and vascular endothelial cells and thus participate in the repair of damaged myocardium. Teng et al. also found that BMSCs markedly enhanced vascularization and blood flow recovery in myocardial infarction by secrete exosomes [14]. Furthermore, Konala et al. found that both endogenous and exogenous BMSCs could leak into the site of inflammation and participate in the local immune regulation process [15]. Other researchers discovered that BMSCs secreted a variety of cytokines; such as vascular endothelial growth factor (VEGF), hepatocyte growth factor (HGF), and basic fibroblast growth factor (bFGF); IL-10; and so on, to decrease apoptosis and fibrosis [16, 17].

We isolated BMSCs and evaluated their effects on a VMC mouse model in vivo. After the murine VMC model had been established, BMSCs were intravenously injected at different stages of VMC. Then the cardiac function was found markedly improved, and the content of col1*α*1, col3*α*1, and TGF-*β* was reduced in the BMSCs therapy group, especially when BMSCs were injected between 1 and 2 weeks after CVB3 inoculation. In conclusion, we found BMSCs ameliorated cardiac fibrosis and the optimal treatment time of BMSCs was 1-2 weeks after CVB3 inoculation.

## 2. Materials and Methods

### 2.1. Animals and Cells

A total of 48 4-week-old male BALB/c mice were obtained from the Shanghai JieSiJie Laboratory Animal Co. Ltd. (Shanghai, China). According to the manufacturer's instructions, BALB/c mouse bone marrow mesenchymal stem cell strains were obtained from Cyagen Biosciences Inc. and cultured with mouse mesenchymal stem cell growth medium (MUCMX-90011, Cyagen Biosciences Inc., Guangzhou, China). The cells were tested, by flow cytometry analysis, positive for CD44 and Sca-1 and negative for CD34 and CD117. Bacteria, fungi, and mycoplasma were also tested negative.

### 2.2. Animal Model and Groups

The 48 inbred male BALB/c mice were randomly divided into the normal control group (control), myocarditis group (VMC), and BMSCs treatment group. Based on different intervention times, the BMSCs treatment group was further randomly divided into four subgroups: VMC1d + BMSCs (intervention on the 1st day after CVB3 inoculation), VMC3d + BMSCs (intervention on the 3rd day after CVB3 inoculation), VMC1w + BMSCs (intervention on the 7th day after CVB3 inoculation), and VMC2w + BMSCs (intervention on the 14th day after CVB3 inoculation). The myocarditis group and BMSCs treatment group were injected intraperitoneally with 50% tissue culture-infective dose (TCID50) of CVB3 (10^9^ Nancy strain, obtained from the Standard Strain Conservation Center in the United States) in 0.2 ml DMEM solution, and the normal control group was injected intraperitoneally with 0.2 ml DMEM solution. The BMSCs treatment subgroups were injected with BMSCs (1 × 10^6^ cells of each mouse with 100 *μ*l PBS) through the tail vein of mice on the 1st day, 3rd day, 7th day, and 14th day after the mice were infected by CVB3. Meanwhile, the normal control group and myocarditis group were injected with 100 *μ*l phosphate-buffered solution through the tail vein.

### 2.3. Echocardiography

Four weeks after CVB3 inoculation, transthoracic echocardiography was performed in all anesthetized mice with the Visual Sonics Vevo 770 instrument, and a 30 MHz high-frequency transducer was used by a blinded investigator. All the echocardiograph measurement data were collected and averaged over three consecutive cardiac cycles.

### 2.4. Myocardial Tissue Pathology

All the mice were sacrificed after 4 weeks of CVB3 infection. Hearts were isolated and made into paraffin-embedded samples, which were sectioned at 5 *μ*m and subsequently stained with hematoxylin and eosin staining (H&E) or Masson's trichrome staining for light microscopy. The analysis of each experimental group was performed with a minimum of 5 replicates of each sample, and 5 visual fields were measured in each replicate. Rezkalla methods were referred to calculate semiquantitative myocardial inflammation pathological scores [18], and the collagen volume fraction (CVF) was calculated by the blue collagen fiber area/the total area of the visual field × 100%.

### 2.5. Quantitative RT-PCR

The total RNA was extracted from cardiac tissue by Trizol reagent (Invitrogen) and reversely transcribed to synthesize cDNA (PrimeScript™ RT Master Mix, Takara). The expressions of col1*α*1 and col3*α*1 mRNA were detected by the SYBR® green real-time fluorescence quantitative method (SYBR Premix Ex Taq™, Takara). The mouse GAPDH, col1*α*1, and col3*α*1 mRNA primers were synthesized by Shanghai Biotechnology Bioengineering Co. Ltd. (Shanghai, China). The primers used were GAPDH (183 bp): 5′-GGT TGT CTC CTG CGA CTT CA-3′ and 5′-TGG TCC AGG GTT TCT TAC TCC-3′, col1*α*1 (117 bp): 5′-TGA CTG GAA GAG CGG AGA GT-3′ and 5′-GAC GGC TGA GTA GGG AAC AC-3′, and col3*α*1 (128 bp): 5′-CGT AAG CAC TGG TGG ACA GA-3′ and 5′-AGC TGC ACA TCA ACG ACA TC-3′. PCR analysis was repeated three times, and their results were presented as the ratio of the target gene to the expression of an internal reference gene.

### 2.6. Western Blot Analysis

The total protein of the myocardium was extracted, and its concentration was determined by the BCA method (Pierce™ BCA Protein Assay Kit, Thermo Fisher Scientific). Tissue protein was separated by 15% SDS-PAGE and then wetted to PVDF membrane (Millipore), 5% BSA closed at room temperature for 2 h, plus anti-primary (1 : 1000 dilution) and incubated overnight at 4°C in a refrigerator, and finally washed and incubated with goat anti-rabbit IgG (Proteintech Group) at room temperature for 2 h. Reactive bands of the PVDF membrane were equipped with ECL developer (Thermo Fisher Scientific) and placed in a BIO-RAD gel imager to image, and the Image Lab software calculated its gray value. The process was repeated three times, and the results were presented as the ratio of the gray value of the target protein band to the GAPDH band. The first antibodies used were monoclonal rabbit anti-Smad2 antibody (Cell Signaling Technology), monoclonal rabbit anti-p-Smad2 antibody (Cell Signaling Technology), monoclonal rabbit anti-TGF-*β*1 antibody (Cell Signaling Technology), and HRP-conjugated monoclonal mouse anti-GAPDH antibody (Shanghai KangChen Bio-tech Co. Ltd., China) (loading control).

Reactive bands were equipped with ECL developer (Thermo Fisher Scientific) which immediately drops on the PVDF membrane, placed in BIO-RAD gel imager imaging and image Lab software to calculate its gray value.

### 2.7. Statistical Analysis

All data were presented as mean ± standard deviation (SD) and subjected to one-way analysis of variance. Tukey's multiple comparison test was used to compare numeric data among the six experimental groups. *P* value < 0.05 was considered as statistically significant.

## 3. Results

### 3.1. Characterization of Experimental Viral Myocarditis

After inoculation of CVB3, the mice showed listlessness or irritability, dull and rough hair, hair loss, weight loss, and occasionally arching back. Mice in the normal control group were generally in good condition and had no abnormal performance. There was no death in the study, and all the mice were included in the analysis.

### 3.2. Bone Marrow Mesenchymal Stem Cells Improved Cardiac Function in Mice with Viral Myocarditis

Four weeks after the mice were being infected by CVB3, transthoracic echocardiographic studies were performed to evaluate heart function of all mice (Table 1). Compared with the normal control group, the left ventricular ejection fraction (LVEF) and fractional shortening (FS) were observably lower in the myocarditis group (*q* = 9.358, 9.417; *P* < 0.05). The thickness of the left ventricular anteroposterior wall attenuated at systolic and diastolic levels; meanwhile, the left ventricular internal diameter (LVIDs) at end-systole and the left ventricular end-systolic volume (LVESV) were significantly increased (*q* = 6.701, 7.052; *P* < 0.05). This indicated that the myocarditis group had dilated cardiomyopathy-like changes. After BMSCs transplantation, the levels of LVEF and FS were significantly higher than those in the myocarditis group (*P* < 0.05), and the VMC1w + BMSCs and VMC2w + BMSCs subgroups had the most significant changes (*q* = 8.748, 8.428, 8.528, 8.190; *P* < 0.01). This suggested that BMSCs could significantly improve the cardiac function after 1-2 weeks of CVB3 infection in mice.

Compared with the myocarditis group, the thickness of the left ventricular anterior wall during systole (LVAWs) was significantly increased in the BMSCs treatment group, and the VMC1w + BMSCs and VMC2w + BMSCs subgroups had the most significant changes (*q* = 6.438, 6.301; *P* < 0.01). The thickness of the left ventricular posterior wall during diastole (LVPWs) was also increased in the VMC1w + BMSCs subgroup compared to that in the myocarditis group (*q* = 4.391; *P* < 0.05). Among all the BMSCs treatment subgroups, the VMC1d + BMSCs subgroup had the lowest level of LVIDs and left ventricular end-systolic volume (LVESV) (*q* = 4.569, 4.373; *P* < 0.05), and the VMC1w + BMSCs and VMC2w + BMSCs subgroups had a much lower level of LVIDs and LVESV than the myocarditis group (*q* = 7.884, 6.783, 7.934, 7.051; *P* < 0.01). But the difference of LVID and LVESV level between the VMC3d + BMSCs subgroup and the myocarditis group was not statistically significant. Furthermore, there was no significant difference in diastolic index between the BMSCs treatment group and the myocarditis group.

### 3.3. Bone Marrow Mesenchymal Stem Cells Reduced Viral Myocarditis-Induced Myocardial Fibrosis

#### 3.3.1. Pathological Changes of the Myocardium after BMSCs Intervention

**Figure 1:**
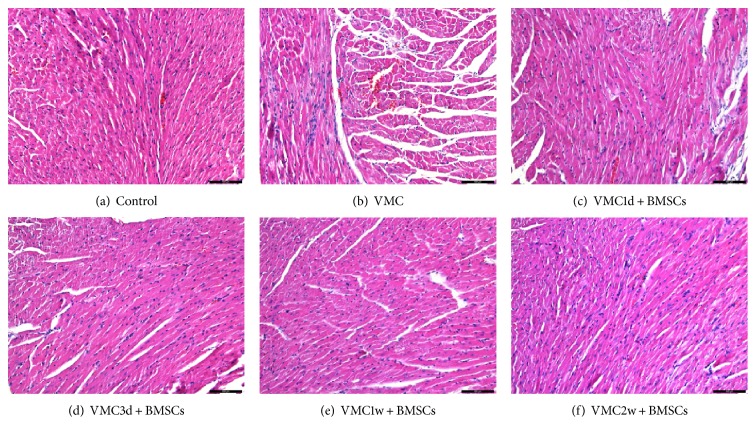
Histological findings of myocardial tissues after BMSCs transplantation. The scale bars are equal to 100 *μ*m in all of the images. (a) Control group. Histological findings of myocardial tissues in 4 weeks after PBS intraperitoneal injection. The myocardium was arranged neatly with clear contours and no obvious inflammatory cell infiltration (H&E, ×200). (b) VMC, the myocarditis group. Histological findings of myocardial tissues within 4 weeks after CVB3 intraperitoneal injection. The myocardium was disordered and showed residual inflammatory infiltration and necrotic lesions (H&E, ×200). (c–f) The BMSCs treatment subgroups. Histological findings of myocardial tissues within 4 weeks after CVB3 intraperitoneal injection. Mice in the VMC1d + BMSCs, VMC3d + BMSCs, VMC1w + BMSCs, and VMC2w + BMSCs subgroups were injected with BMSCs (1 × 10^6^ cells of each mouse with 100 *μ*l PBS) into their tail vein on the 1st day, 3rd day, 1st week, and 2nd week after they were infected by CVB3, respectively. The myocardium showed clear contours and no obvious inflammatory cell infiltration (H&E, ×200).

**Figure 2:**
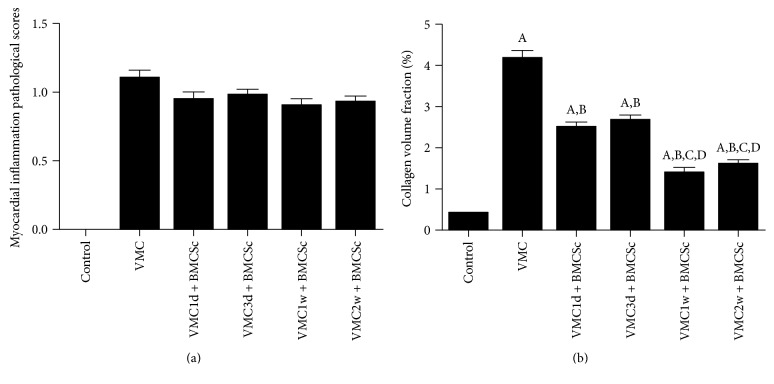
(a) The figure presented the myocardial inflammation pathological scores in different groups and subgroups. The difference among the groups was not statistically significant. (b) The collagen volume fraction of myocardial tissues. ^A^*P* < 0.05 when compared to the control group; ^B^*P* < 0.05 when compared to the VMC group; ^C^*P* < 0.05 when compared to the VMC1d + BMSCs subgroup; and ^D^*P* < 0.05 when compared to the VMC3d + BMSCs subgroup.

H&E staining showed that the myocardial cells in the normal control group were neatly arranged with clear contours, and no infiltration of inflammatory cells was observed in the myocardium. After 4 weeks of CVB3 infection, there were residual inflammatory infiltration and necrotic lesions in the myocardial tissue of the myocarditis group. The BMSCs treatment group had no obvious inflammatory cell infiltration in the myocardium, and the arrangement of cardiomyocytes was neater than that in the myocarditis group (Figure 1). The myocardial pathologic score in the myocarditis group was 1.1 ± 0.378, and those in the BMSCs treatment subgroups were 0.95 ± 0.3162, 0.97 ± 0.2762, 0.9 ± 0.3038, and 0.925 ± 0.2667. The comparisons were not statistically significant between two or more groups of figures (Figure 2(a)).

**Figure 3:**
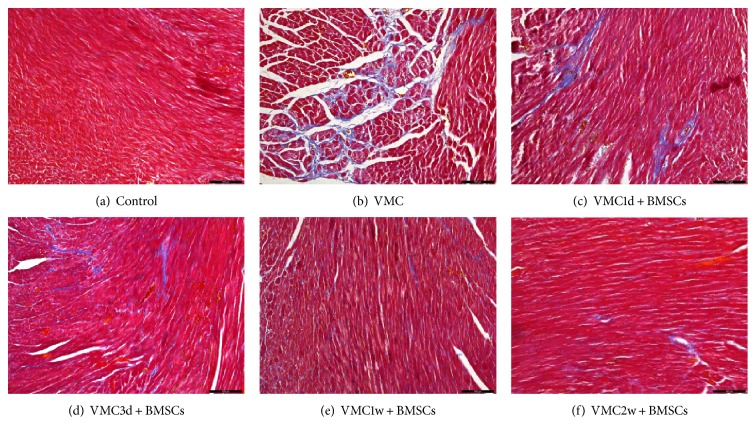
(a–f) Myocardial tissue collagen (blue) fiber staining results (Masson's trichrome staining, ×200, scale bar = 100 *μ*m). (a) Control group. Histological findings of myocardial tissues in 4 weeks after PBS intraperitoneal injection. (b) VMC, the myocarditis group. Histological findings of myocardial tissues within 4 weeks after CVB3 intraperitoneal injection. (c–f) The BMSCs treatment subgroups. Histological findings of myocardial tissues within 4 weeks after CVB3 intraperitoneal injection. Mice in the VMC1d + BMSCs, VMC3d + BMSCs, VMC1w + BMSCs, and VMC2w + BMSCs subgroups were injected with BMSCs (1 × 10^6^ cells of each mouse with 100ul PBS) through their tail vein on the 1st day, 3rd day, 1st week, and 2nd week, respectively, after they were infected by CVB3.

Masson's trichrome staining showed that different degrees of blue fibrous tissue hyperplasia were observed in the myocardium of the myocarditis group and the BMSCs treatment group. Besides, the BMSCs treatment group had less fibrous tissue hyperplasia than the myocarditis group, especially in the VMC1w + BMSCs and VMC2w + BMSCs subgroups (Figure 3). The CVF in the myocarditis group was higher than those in all the BMSCs treatment groups (*F* = 98.09; *P* < 0.001). Among the BMSCs treatment subgroups, the CVF in the VMC1w + BMSCs and VMC2w + BMSCs subgroups were lower than those in the VMC1d + BMSCs and VMC3d + BMSCs subgroups (Figure 2(b)).

#### 3.3.2. Bone Marrow Mesenchymal Stem Cells Reduced the Expression of col1*α*1 and col3*α*1 mRNA in Viral Myocarditis

**Figure 4:**
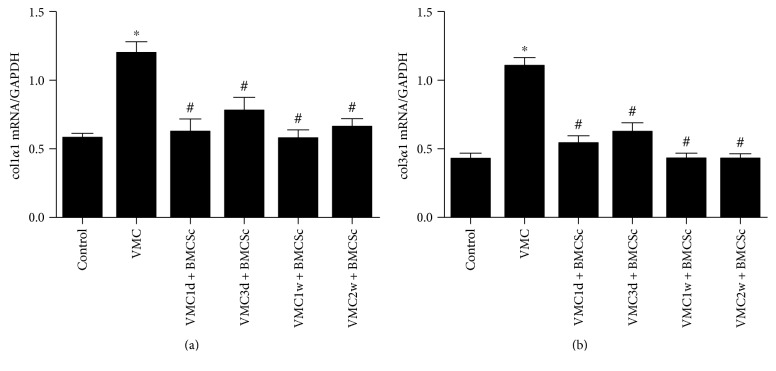
Quantification of col1*α*1 and col3*α*1 mRNA levels. (a) The relative expression of col1*α*1 mRNA in myocardial tissue. In 4 weeks after injection of CVB3, the expression of col1*α*1 mRNA was higher in the VMC group than in the BMSCs treatment subgroups. (b) The relative expression of col3*α*1 mRNA in myocardial tissue. The expression of col1*α*1 mRNA was higher in the VMC group than in the BMSCs treatment subgroups. *n* = 8 for each group. ^∗^*P* < 0.05 when compared to the control group; ^#^*P* < 0.05 when compared to the VMC group.

The expression of col1*α*1 and col3*α*1 mRNA was significantly higher in the myocarditis group than that in the normal control group and the BMSCs treatment group (*F* = 9.962, 25.79; *P* < 0.05). There was no significant difference between the normal control group and the BMSCs treatment group (Figure 4).

### 3.4. Bone Marrow Mesenchymal Stem Cells Reduced the Expression of TGF-*β*1 Protein in Viral Myocarditis

**Figure 5:**
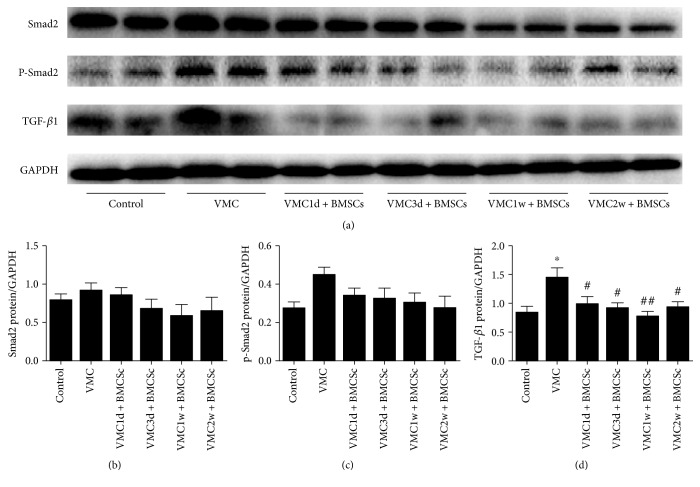
The expression of the TGF-*β*1 signaling pathway related to protein in the myocardium with BMSCs treatments. (a) The expression levels of Smad2, p-Smad2, and TGF-*β*1 in the myocardium were analyzed by Western blotting. (b–d) Quantitative data of Smad2, p-Smad2, and TGF-*β*1 in the myocardium. *n* = 8 for each group. ^∗^*P* < 0.05 when compared to the control group; ^#^*P* < 0.05 when compared to the VMC group; and ^##^*P* < 0.01 when compared to the VMC group.

Western blot was used to detect the expression of Smad2, p-Smad2, and TGF-*β*1 protein in each group (Figure 5(a)). Compared with the myocarditis group, the expression of Smad2 and p-Samd2 protein tended to decrease in the BMSCs treatment group, but the difference between the two groups was not statistically significant (Figures 5(b) and 5(c)). The gray value ratio of TGF-*β*1 protein band and GAPDH band was significantly lower in both the normal control group and the BMSCs treatment group than in the myocarditis group (*F* = 4.317; *P* < 0.05), especially in the VMC1w + BMSCs subgroup (*q* = 6.085; *P* < 0.01), and there was no significant difference between the normal control group and the BMSCs treatment group (Figure 5(d)).

## 4. Discussion

VMC may develop into DCM due to cardiac fibrosis and ultimately lead to heart failure [19]. In the restoration and chronic stage of VMC, the fibrosis hyperplasia was more obvious [20]. Therefore, reducing the excessive proliferation of fibrous tissue in viral myocarditis at an appropriate intervention time is necessary for preventing DCM and heart failure [21]. In the present study, we demonstrated that BMSCs ameliorated cardiac fibrosis and cardiac dysfunction following VMC in a murine model and we, for the first time, use BMSCs to treat VMC murine model in different stages of this disease. Finally, we demonstrated that the optimal intervention time was 1-2 weeks after CVB3 inoculation. In the myocarditis group, we found that myocardial inflammation, during a 28-day follow-up period, significantly subsided, but excess growth and proliferation of cardiac fibroblasts were increasingly severe. Masson's trichrome staining showed a significant increase of collagen fibers in the myocardial interstitium, and the cardiomyocytes were arranged in disorder. The expression of type I and type III collagen fibers which were abundant in the myocardium increased most significantly. Moreover, echocardiographic screening indicated that the left ventricular wall thickness was greater compared with that in the normal control group and the left ventricular end-systolic diameter increased. Besides, the LVEF and FS decreased significantly, which indicated that sustained increase of collagen fibers in the myocardium may cause damage to its cardiac function. After treating with BMSCs, collagen fibers significantly decreased and cardiac function was improved. Furthermore, collagen fibers decreased even more in both the VMC1w + BMSCs and the VMC2w + BMSCs subgroups than in the VMC1d + BMSCs and VMC3d + BMSCs subgroups. Echocardiography also showed that the LVEF and FS in the VMC1w + BMSCs and VMC2w + BMSCs subgroups were higher than those in the VMC1d + BMSCs and VMC3d + BMSCs subgroups, which suggested that the reduction of myocardial fibrosis may contribute to the improvement of cardiac function. And the lesser the collagen fibers are, the more improvement of cardiac function there may be.

BMSCs, derived from bone marrow, can reduce the synthesis of collagen fibers, but the mechanisms are still under discussion. Chen et al. discovered that BMSCs alleviated fibrosis by secreting a variety of cytokines such as hepatocyte growth factor (HGF) [22], and Ishikawa et al. found that it is the downregulation of TGF-*β*1 expression that alleviates fibrosis in different diseases [23]. Mias et al. uncovered that, when myocardial fibroblasts are activated, a great deal of metal matrix protease will be secreted and consequently ameliorates the formation of myocardial fibrosis [24]. Besides, in cardiovascular disease, transplantation of BMSCs can inhibit apoptosis after myocardial infarction [25]. BMSCs can also differentiate into cardiomyocytes and vascular endothelial cells to treat myocardial necrosis and increase local blood supply [26]. Wang et al. demonstrated that with a strong plasticity, BMSCs can perform various functions in accordance with the microenvironment [27]. For example, the types and intensity of inflammation in different microenvironments may determine which kind of function BMSCs will perform, proinflammatory or anti-inflammatory [28]. Although BMSCs have been studied by many scholars, there is currently no published standard for BMSCs transplantation. And few researches have focused on the therapeutic effect and optimal transplantation time of BMSCs in VMC. Zhao et al. [29] have studied stem cell mobilization in a VMC murine model and found that the optimal transplantation time may be within 2 weeks after viral infection, but the study did not tell us the optimal transplantation time to reduce fibrosis.

Myocardial fibrosis is a chronic and progressive process characterized by an excessive accumulation of extracellular matrix (ECM) [30] and is regulated by a variety of factors, among which TGF-*β*1 is the most important one [31]. TGF-*β*1 is a member of the transforming growth factor beta superfamily and a secreted protein that is recognized as a regulator of cell growth, cell proliferation, cell differentiation, and apoptosis. In particular, TGF-*β*1 can promote fibroblast proliferation and the synthesis of extracellular matrix (ECM), participate in endothelial stromal transformation (EndMT), and inhibit the degradation of ECM [32]. Consistent with the findings by Guo et al. [33], we found that the expression of the TGF-*β*1 protein in the myocarditis group was significantly higher than that in the normal control group and was significantly decreased in the BMSCs treatment groups. And the further studies of Sun et al. conclusively showed that sustained expression of TGF-*β*1 can promote the formation of collagen and the growth of fibroblasts [34]. In our study, the expression of Smad2 and p-Smad2 protein in the BMSCs treatment subgroups tended to decrease, but there was no significant difference when compared to those in the normal control group and myocarditis group. These evidences suggested that BMSCs may reduce the expression of TGF-*β*1 to ameliorate myocardial fibrosis, but this effect may not entirely depend on the p-Smad2 signaling pathway. This is in contrast to the findings by Chen et al. [35], who discovered that ameliorated myocardial fibrosis was in line with the reduced expression of TGF-*β*1 and its downstream p-Smad2 in the myocardium.

In conclusion, the transplantation of BMSCs can reverse the damage caused by viral myocarditis and reduce the formation of myocardial fibrosis in a VMC murine model. And the optimal intervention time of BMSCs may be 1-2 weeks after CVB3 inoculation in a VMC murine model. These results provide a potential therapeutic strategy for the treatment of VMC, but it still needs more studies to discover the mechanisms and ensure its application in human beings.

## Figures and Tables

**Table 1:** Echocardiography on the 28th day after CVB3 inoculation. *n* = 8 for each group.

Parameters	Control	VMC	VMC1d + BMSCs	VMC3d + BMSCs	VMC1w + BMSCs	VMC2w + BMSCs
Heart rate (beats/min)	509 ± 31	521 ± 30	531 ± 39	535 ± 14	533 ± 16	529 ± 31
LVAWd (mm)	0.736 ± 0.058	0.557 ± 0.079^aa^	0.597 ± 0.031^a^	0.593 ± 0.086^a^	0.610 ± 0.039^a^	0.648 ± 0.113
LVAWs (mm)	1.210 ± 0.102	0.735 ± 0.077^aa^	0.960 ± 0.100^a,b^	1.036 ± 0.178^bb^	1.029 ± 0.095^bb^	1.023 ± 0.179^bb^
LVPWd (mm)	0.721 ± 0.106	0.546 ± 0.068^a^	0.592 ± 0.047	0.590 ± 0.089^a^	0.611 ± 0.066	0.645 ± 0.120
LVPWs (mm)	1.125 ± 0.153	0.811 ± 0.120^aa^	0.952 ± 0.116	0.923 ± 0.130	1.029 ± 0.139^b^	1.010 ± 0.172
LVIDd (mm)	3.684 ± 0.255	3.941 ± 0.366	3.593 ± 0.387	3.645 ± 0.267	3.440 ± 0.459	3.466 ± 0.410
LVIDs (mm)	2.149 ± 0.300	2.970 ± 0.375^aa^	2.410 ± 0.459^b^	2.600 ± 0.221	2.004 ± 0.328^bb,c^	2.139 ± 0.349^bb^
LVEDV (*μ*l)	55.61 ± 13.22	68.40 ± 14.61	55.03 ± 13.93	56.57 ± 9.30	50.02 ± 15.39	50.74 ± 14.41
LVESV (*μ*l)	15.79 ± 5.62	34.93 ± 10.77^aa^	21.53 ± 10.41^b^	24.90 ± 5.09	13.39 ± 5.32^bb,c^	15.79 ± 6.59^bb^
LVEF (%)	71.98 ± 5.149	49.55 ± 6.999^aa^	62.41 ± 9.749^b^	60.22 ± 6.673^b^	70.51 ± 6.166^bb,c^	69.74 ± 4.763^bb^
FS (%)	40.59 ± 4.303	24.81 ± 4.145^aa^	33.33 ± 6.554^b^	31.74 ± 4.617	39.10 ± 4.71^bb,c^	38.53 ± 3.54^bb^

LVAWs: left ventricular anterior wall during systole; LVAWd: left ventricular anterior wall during diastole; LVPWs: left ventricular posterior wall during systole; LVPWd: left ventricular posterior wall during diastole; LVIDs: left ventricular internal diameter at end-systole; LVIDd: left ventricular internal diameter at end-diastole; LVESV: left ventricular end-systolic volume; LVEDV: left ventricular end-diastole volume; LVEF: left ventricular ejection fraction; FS: fractional shortening). ^a^*P* < 0.05 when compared to the control group; ^aa^*P* < 0.01 when compared to the control group; ^b^*P* < 0.05 when compared to the VMC group; ^bb^*P* < 0.05 when compared to the VMC group; and ^c^*P* < 0.05 when compared to the VMC3d + BMSCs group.
